# Torsional Vibrations in the Resonance of High-Speed Rotor Bearings Reduced by Dynamic Properties of Carbon Fiber Polymer Composites

**DOI:** 10.3390/ma16093324

**Published:** 2023-04-24

**Authors:** Zuzana Murčinková, Jozef Živčák, Dominik Sabol

**Affiliations:** 1 Department of Design and Monitoring of Technical Systems, Faculty of Manufacturing Technologies with a Seat in Prešov, Technical University of Košice, Bayerova 1, 080 01 Prešov, Slovakia; dominik.sabol@tuke.sk; 2 Department of Biomedical Engineering and Measurement, Faculty of Mechanical Engineering, Technical University of Košice, Letná 9, 042 00 Košice, Slovakia; jozef.zivcak@tuke.sk

**Keywords:** cage slip, fluctuating pressing load, drop in rotational speed, hierarchical design, carbon chopped fibers and nanofibers, passive damping, polymer composite materials, macroshapes

## Abstract

The present study deals with the harmful torsional resonance vibrations of textile rotor bearings, the amplitudes of which are reduced mainly by the use of high-capacity damping materials, characterized by an internal hierarchical structure and macroshape, added into the machine mechanical system. The additional materials are polymer matrix composites reinforced either by carbon nanofibers or carbon chopped microfibers and either aramid or carbon continuous fibers. The macroshape is based on a honeycomb with internal cavities. Torsional vibrations arise in mechanical systems as a result of fluctuations in the low-level pressing load of the flat belt driving the rotor-bearing pin and the changing of kinematic conditions within it, which, in the resonance area, leads to cage slip and unwanted impulsive torsional vibrations. Moreover, this occurs during high-frequency performance at around 2100 Hz, i.e., 126,000 min^−1^. The condition, before the redesign, was characterized by significantly reduced textile rotor-bearing life due to significant impulse torsional vibrations in the resonance area. The study showed a significant reduction in average and maximum torsional amplitudes in the resonance area by 33% and 43%, respectively. Furthermore, the paper provides visualization of the propagation of a stress wave at the microscale obtained by the explicit finite element method to show the dispersion of the wave and the fibers as one of the sources of high damping.

## 1. Introduction

Torsional vibrations in the machine unit, which are the subject of this article, are induced during operation in the resonance area of the system from the slippage of the specific textile high-speed rotor rolling bearing cage. A rolling bearing cage is a component exposed to complex load variations under different operating conditions. The condition of high-speed rotation with low-level radial load causes insufficient contact force between the inner ring and the roller, which results in cage and roller slip and subsequently leads to skidding [1,2]. Slip occurs when the radial load is inadequate to develop frictional (tractional) force between rolling elements and the rotating raceway to overcome drag forces [2]. Skidding due to extreme sliding between the rolling elements and raceway causes wear on the rolling contact surfaces and results in the smearing type of surface damage [3]. The wear on the inner rolling track and elements is excessive and leads to damage to the bearing and premature failure before it has reached the fatigue life. According to our measurements, premature failure appears within about 1000 h of operation.

The effect of operating parameters, namely shaft speed, radial load, viscosity of lubricating oil, number of rollers, and bearing temperature, on cage slip have been obtained experimentally [3]. Moreover, the irregular variation of the rotational speed of the roller produces a frequent impact on the cage, which causes the cage to suffer violent collision force [4]. Furthermore, in [1], the cage slip ratio was quantified according to clearance changes in various operating conditions and geometric parameters, and the causes of cage slip were analyzed using the simplified dynamic model. An experimental and simulation study [5] describes the effective solution of the light-load slip in terms of the deformation of cylindrical roller bearing rings due to machining principles, resulting in a trilobe (non-circular) raceway.

Owing to the aforementioned cage slip, the shaft does not always rotate with a constant angular velocity, and torsional vibrations of the elastic shaft of the textile rotor bearing are generated. Such vibrations are typical of gearbox operation, drilling, milling, etc. Excessive torsional vibration in the machine system leads to noise or fatigue destruction [6].

A preferable option is to reduce the source(s) of the harmful torsional vibrations, which has also been our approach to reducing the impulse torsional vibrations. The response of mechanical systems can be controlled by passive, active, and semi-active dampers. Various passive methods to suppress vibrations have been discussed and studied in the literature. Passive vibration reduction is based on mechanical effects such as damping, absorption, isolation, or neutralization. However, the damping of torsional vibration is unique because of the rotational character of the motion, and the literature on torsional vibration elimination in rotating systems is very limited. The attached-ball balancer, centrifugal pendulum absorber, roller-type centrifugal absorber [7], and passive mass-spring-disc dynamic vibration absorber involving springs [6] are devices designed to eliminate the torsional vibrations of a rotating shaft. Numerous passive systems have been proposed by researchers; most famous among them, perhaps, are friction control devices, fluid viscous dampers, seismic base isolators, tuned liquid dampers, and tuned mass dampers [8]. Passive systems dissipate a portion of the structural input energy, thus reducing the structural response and possible structural damage [8] with no external power.

The dampers used to absorb the torsional vibration are generally designed using rubber or viscous dampers for high torque and high horsepower engines [9]. In [10], the peak amplitude of torsional vibrations of the turbo-generator rotor was reduced by 60% using passive and semi-active magnetorheological dampers. Applied to the workpiece-holding system during milling, tuned viscoelastic dampers reduce vibration amplitudes by up to 98% in the target mode range and by up to 53% in the non-target mode range. A linear viscous damper at optimal tuning can increase the flutter critical rotational speed of wind turbine blades by more than 100% [11]. The results obtained in [9] showed that a hybrid design with an optimized double-mass rubber and viscous torsional vibration damper reduced the torsional vibrations by 50.17% at the crankshaft end and by 79.65% on the pulley when compared to the non-optimized one.

In recent decades, material development has focused on the fulfillment of the unusual combination of properties, e.g., high stiffness–high loss coefficient, low density–high strength, low density–high stiffness, low density–high fracture toughness, high strength–low stiffness, high temperature–high strength, etc. According to [12], there are three approaches to obtain unusual mechanical, thermal, electrical, etc., properties. The methods are based on (i) manipulating chemistry, (ii) manipulating microstructure, using thermomechanical processing, and (iii) controlling the architecture to create hybrid materials. The third approach was applied in the present study to obtain a stiff and highly damped material design. Researchers have investigated the possibility of controlling the architecture and topology of components through the hierarchical lattice metamaterial structure [13], novel architecture microstructure [14], and optimization of their topology [15,16]. Meanwhile, the problem of manufacturability of these structures arises, and 3D printing becomes a successful technology in this regard. However, even 3D printing as a “boom” technology has its own problems that need to be overcome, such as determining the material properties of components printed in this way [17], the influence of configuration parameters [18,19], and specific design for 3D printing [19].

The development of new materials has brought some new opportunities. Fiber polymer composites have unique and excellent mechanical properties, fatigue resistance, and corrosion resistance, which further expand their application fields. With the combination of different functions, important multifunctional carbon-fiber-reinforced polymer composites and other materials can be created for the energy and transportation industry, autonomous vehicles, and Industry 4.0 [20,21,22].

In this work, high-speed operation within the resonance range led to a sudden de-acceleration and acceleration due to cage slip and the accompanying effects of beating operation and the appearance of torsional vibration. Moreover, in our case, the torsional conditions are enhanced by fluctuation of the pressing load of the driving belt. The present study deals with the change in the dynamic properties of the components, using the mainly passive damping of polymer composites. Furthermore, additional high-damping materials containing composite nanofibers, in three macrolayers and a specific macroshape with a hierarchical carbon-based structure, influence not only the damping and the stiffness, but also the modal properties of the individual components and the entire mechanical system. Changing the modal properties of components using shape and/or dimensions is also used to reduce vibration amplitudes. The rotor bearing casing and pressure plate of the idler pulley for the driving belt are components chosen mainly for their ability to increase material damping in order to reduce or prevent the generation of cage slip and torsional vibration in the resonance range. The most important thing is to ensure safe, and not shortened, bearing operation. Whereas other studies are limited to focusing on cylindrical rolling bearings and the damping of torsional vibrations in rotating systems using fluids and rubber, our study focuses on the housing of ball rotor bearings, pressure plates, and composites to solve the issue.

## 2. Methods and Materials

### 2.1. Components and Materials

**Figure 1:**
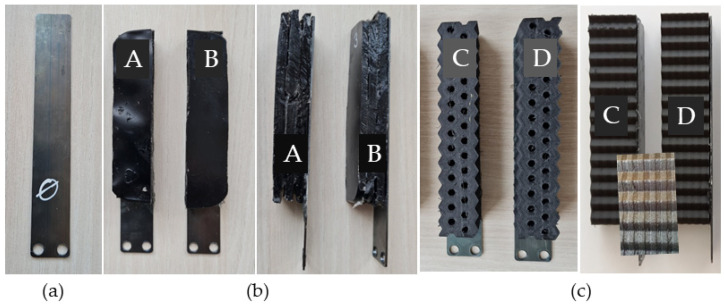
Tested pressure plates: (**a**) Original PP_0; (**b**) top and right view of PP_A and PP_B with polymer composite filled with nanofibers in three macrolayers; (**c**) top and right view PP_C and PP_D with polymer composite of specific macroshape with a hierarchical carbon-based structure (the layers in detail).

A comparison of the dynamic response of the mechanical system using amplitude vibration during the step-by-step replacement of the original and redesigned components was made. We compared the original (Figure 1a) and four types of pressure plates (PPs) for damping the idler pulley of the flat driving belt. The tested pressure plates in Figure 1b,c were redesigned using the composite materials that were attached to the original steel pressure plate, PP_0, with dimensions 130 × 20 × 1 mm (length × width × thickness) and 2 holes, 5 mm in diameter.

The composite materials attached to the original steel plate were based on carbon fibers placed in a polymer matrix (epoxy resin L 285 and curing agent MGS LH285, produced by Satria, Olomouc, Czech Republic). The redesigned pressure plates, PP_A and PP_B (Figure 1b, Table 1), included a polymer composite of dimensions 95 × 20 × 15 mm in three macrolayers with a thermosetting epoxy resin matrix and 1 wt% of multi-walled carbon nanotubes (MWCNTs) produced by Sigma-Aldrich, Saint-Louis, Missouri, USA. The dimensions of the nanotubes for PP_A and PP_B were significantly different, which is expressed by the ratio of nanotube diameter to its length, i.e., the aspect ratio in Table 1. MWCNTs in PP_A had outer diameter 6–13 nm, average diameter 8.7–10 nm, inner diameter 2–6 nm, length 2.5–20 µm, average length 10 µm, surface area 216 m^2^/g, and density ~2.1 g/mL at 25 °C. For MWCNTs, available parameters are diameter 110–170 nm, length 5–9 µm, and density ~1.7–2.1 g/mL at 25 °C.

Pressure plates PP_C and PP_D were based on a polymer composite of dimensions 110 × 20 × 25 mm and Onyx material (produced by Markforged, Waltham, MA, USA) as a nylon-based thermoplastic with short, chopped strands of carbon fibers. For the multiscale hierarchic inner microscale architecture of the PP_C and PP_D pressure plates, continuous aramid (Kevlar) and carbon fibers, respectively, were added in layers to the original nylon matrix along with chopped carbon fibers (Table 1). Moreover, the macroscopic structure of PP_C and PP_D was based on a honeycomb shape, with hexagonal holes. These PPs also contained inner cavities. All redesigned pressure plates had a maximum mass difference of 4.6%. The basic mechanical parameters of constituents for the preparation of PP_A-PP_D are in Table 2.

**Figure 2:**
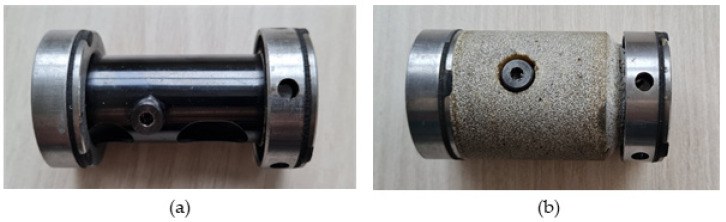
Tested rotor casings: (**a**) original RC_0; (**b**) RC_3 with polymer composite filled with silica sand.

For the measurements, we used two types of rotor casing (RC) (Figure 2, Table 3) for each pressure plate. Rotor casing RC_0, marked as original (Figure 2a), had steel bushings at its ends filled with rubber. Rotor casing RC_3 was made of original rotor casing enriched by additional material made from 70 wt% silica particles in an epoxy resin matrix. That polymer–concrete composite increases the mass of the central part of the RC by 52%, as well as increasing the along with stiffness and material damping.

### 2.2. Experimental Equipment and Method

When the rotational speed increases, a resonance effect arises in the mechanical system under investigation. In measurements with 11 rotor bearings in the spinning units of the textile machine driven by the same flat belt, the response of each individual bearing is different. Certain rotor bearings in the resonance area manifest slippage and subsequent accompanying effects. For the present investigation, we focused on an operation with this type of bearing response within the resonance range. The resulting effects of slippage are as follows:

- Torsional vibrations;
- A sharp drop in rotational speed and a large slippage in the bearing causing other adverse degradation effects;
- Increased pulsating stress on the bearing cage and a decrease in the lifetime of the cage;
- Large and significant changes in the friction moment of the bearing;
- Increased friction between the driving belt and the rotor pin;
- Friction between the driving belt and the pin resulting in heating of the pin;
- The temperature increase changing the clearance in the bearing.

As a result of these phenomena, wear and micropitting occur on the pin in a short period of 1000 h.

**Figure 3:**
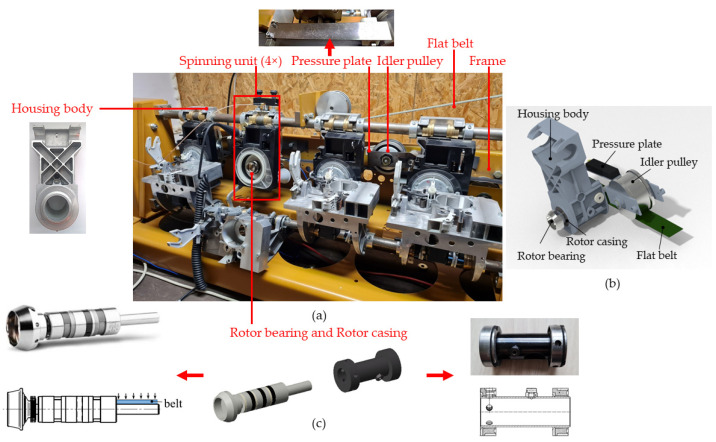
(**a**) Test bench; (**b**) CAD model of principle components of the mechanical system, (**c**) rotor bearing (left) and rotor casing (right).

The aim of the investigation was to reduce the dynamic properties of modified components of a textile test bench using composite structures for the elimination of the above phenomena. We focused on finding out how these changed properties affect the operation of the rotor bearing by measuring the impulsive amplitudes of the torsional resonance vibrations. We used a test bench (Figure 3a)—a model of a textile machine in the test room of Technická Diagnostika, s.r.o. (Technical Diagnostics, Ltd., Prešov, Slovak Republic, https://www.diagnostika.sk/en/) (accessed on 12 February 2023), the cooperating company with the Technical University of Košice.

The test bench comprised four spinning units (Figure 3a) consisting of a rotor housing body and a high-speed rotor bearing driven on its end-pin by a flat belt (Figure 3c, left). The rotor bearing was housed in a rotor casing (RC) (Figure 3c, right), which consisted of a steel thin-walled tube with steel bushings at its ends. The rotor bearing and rotor casing components were both housed in the rotor housing body, which was mounted to the rod of the test bench frame. The rod represents the rod of the real textile machine line. While the high-speed rotor bearing was rotating, the rotor housing body and rotor casing were static. A flat driving belt was in contact with the idler pulley, which was damped by a pressure plate (PP) mounted by two screws on the test bench frame.

The high-speed rotor bearing was not a standardized bearing. The rotor consisted of an outer case, two separate rows of rolling elements (balls) in cages, and a shaft, one end-pin of which was adapted for driving by the flat belt with the other end-pin for the cup head (Figure 3c, left). The outer diameter and length of the rotor bearing were 22 mm and 120 mm, respectively, and the pin diameter was 8.5 mm. The radial clearance is set by the producer and its prescribed range was 4 to 8 μm. The precision grade of the high-speed rotor was 5–10 times higher than standard bearing production.

The following dynamic parameters were measured:

- Absolute vibrations on the rotor body—acceleration (ACC) amplitudes (g) in the frequency range up to 60 kHz;
- Acoustic emission (V) in the frequency range up to 200 kHz.

**Figure 4:**
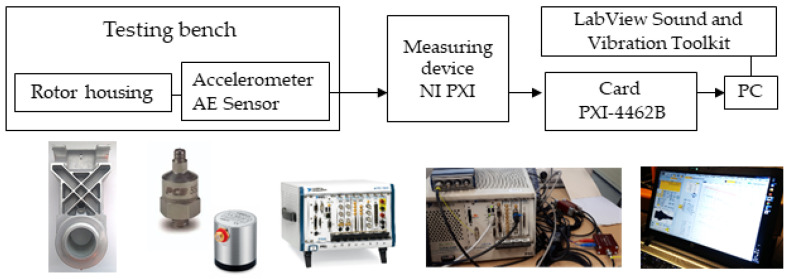
Measurement setup.

The experimental study was carried out on the test bench (Figure 3a) using the measurement setup in Figure 4. Absolute vibrations were measured in accordance with standard ISO 10816-3: Mechanical vibrations (https://www.iso.org/standard/50528.html, accessed on 12 February 2023).

The measurement setup involved the following (Figure 4):

- A PCB model 352A60 accelerometer (PCB Piezotronics, Depew, NY, USA), with a frequency range of up to 65 kHz and a sensitivity of 10 mV/g;
- A Vallen-VS45-H acoustic emission sensor with a range of 20–400 kHz;
- A PXI-4462 dynamic signal acquisition device and an NI PXI Sound and Vibration Module Meter (National Instruments Corporation, Austin, TX, USA);
- An A/D converter with 24-bit resolution and a sample rate, in samples-per-second, of 1 kS/s to 204.8 kS/s in 181.9 μS/s increments;
- LabView Sound and Vibration Toolkit software for the advanced analysis of the dynamic signal base (National Instruments Corporation, Austin, TX, USA).

## 3. Results

### 3.1. Original Dynamic Response

**Figure 5:**
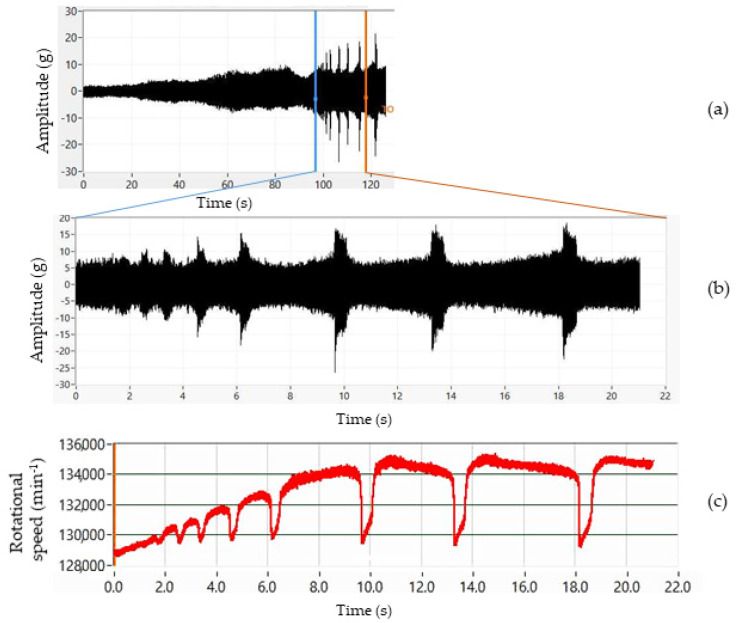
(**a**) Response of original design. ACC amplitudes dependence on the rotational speed between 75,000 up and 135,000 min^−1^; (**b**) detail; (**c**) drops in rotational speed (max. of 6000 min^−1^).

It should be emphasized that the particularly high torsional vibration amplitudes and the cage slippage phenomenon do not occur for every rotor in the resonance range of the mechanical system. That large additional excitation is visible in the time records shown in Figure 5a,b and manifests in high impulses caused by cage slip in the dynamic signal. While smoothly increasing the operating speed from 75,000 to 135,000 min^−1^ over 120 s (Figure 5a), the rotational speed and the force on the cage change periodically, and the operating conditions can be regarded as mostly stable up to 125,000 min^−1^. However, for the tested bearings at speeds above 129,000 min^−1^, the short intervals with high impulse amplitudes and drops in rotor speed (Figure 5c) show the unfavorable dynamic characteristics of the original state dynamic response. The rotor speed drop is up to 6000 min^−1^ within 0.03 s.

**Figure 6:**
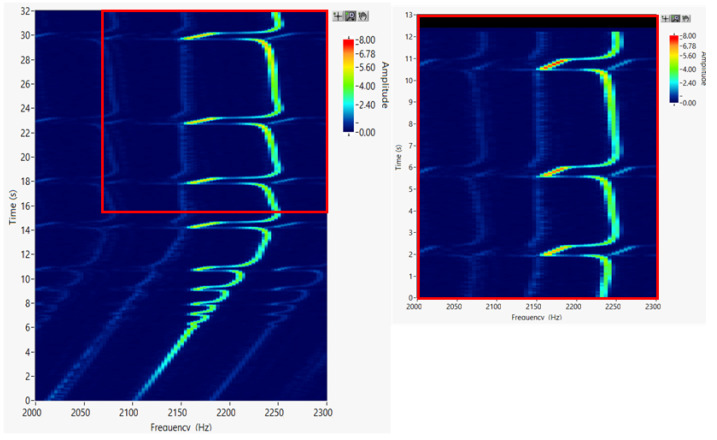
Waterfall plot with detail (a calculation step of 0.01 s); rotational frequency (Hz) vs. time (s) vs. ACC amplitude (g).

The mechanical system produces a non-stationary vibration response in the form of drops in rotational speed even at constant rotational speed. The representation of the spectrum over time is expressed by the waterfall method in Figure 6, also known as short-time Fourier transform or spectrogram. Figure 6 illustrates the decrease in rotational frequency with time at a constant rotational speed of 134,800 min^−1^ and the corresponding amplitude.

The high peaks of the measured dynamic signal are caused by sudden acceleration and deceleration causing torsional deformation. Owing to the elasticity of the pin material, high amplitude torsional vibrations and a sudden increase in kinematic slip are generated. Thus, the frictional moment of the rotor bearing is large, i.e., above the limit value, and the driving belt is not able to transmit the load. This results in slippage of the driving belt on the rotor pin, overheating of the pin-belt contact, and rapid wear of the pin surface. Slippage is acoustically manifested by a significant whistling of the rotor bearing that is repeated regularly.

**Figure 7:**
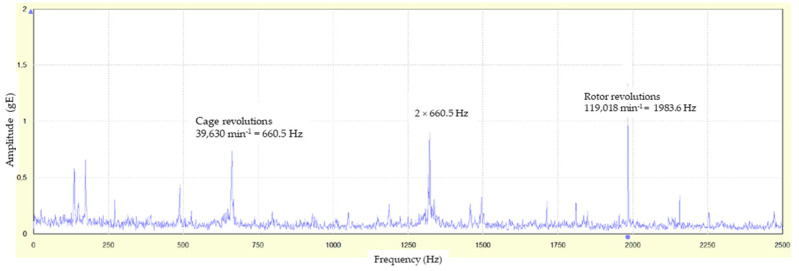
FFT spectrum to determine the actual rotor cage revolutions.

These impulses arise in conditions of cage slip. In the experiment, the cage slip caused by the impact between the cage and balls was detected by analyzing the FFT (fast Fourier transform) spectrum of the dynamic signal (Figure 7). Methods based on the Fourier transform are useful for the analysis of the nonstationary signals and for avoiding the loss of fault information. Involving the convolutional neural network, the bearing life can be effectively predicted [23,24].

The epicyclic values of the angular velocity of the cage, *ω_c_*, for the pure rolling condition can be obtained, for various shaft speeds, using the kinematic equation (according to [3])

(1) $$\omega_{c}=\frac{1}{2(1+r/R_{1})}\omega_{i}$$

where *r* is the radius of the rolling element, *R*_1_ is the radius of the inner race, and *ω_i_* is the angular velocity of the inner race. The percentage of cage slip is derived in terms of kinematics and measured values as follows:

(2) $$\%\;\mathrm{of}\;\mathrm{cage}\;\mathrm{slip}=(1-\frac{\omega_{c,\;\mathrm{measured}}}{\omega_{c,\;\mathrm{kinematic}}})\times 100\%=(1-\frac{n_{c,\;\mathrm{measured}}}{n_{c,\;\mathrm{kinematic}}})\times 100\%=(1-\frac{f_{c,\;\mathrm{measured}}}{f_{c,\;\mathrm{kinematic}}})\times 100\%$$

where *ω_c_*, *n_c_*, and *f_c_* are cage angular velocity, revolutions, and rotational frequency, respectively.

The measured angular velocity is obtained from the FFT spectrum of the dynamic signal (Figure 7). In Figure 7, the constant measured revolutions of the shaft are *n_s_* = 119,018 min^−1^ (peak 1983.6 Hz), and the corresponding kinematic revolutions of the cage should be *n_c_*_, kinematic_ = 42,680 min^−1^. However, the measured revolutions of the cage in Figure 7 are *n_c_*_,measured_ = 39,630 min^−1^ (peaks 660.5 Hz, 2 × 660.5 Hz = 1321 Hz). Using Equation (2), the cage slip is 7.15%, which is a high degree of slip, above the limit value of 0.3%.

Initial measurements confirmed the following:

- The pressure of the flat belt on the rotor pin is not constant and changes with a frequency of 14 Hz;
- The pressing load of the driving flat belt fluctuates according to the frequency of the pulley and the pressure plate in the same cycles;
- The radial pressing force of the flat belt is small, and the nominal value is 12 N ± 2 N, which contributes to the cage slip conditions;
- The rotor rotates at a variable angular velocity, and the rotating of the shaft is enriched with rotational speed drops in the resonance range, the drops corresponding with the ACC amplitude peaks (Figure 5 and Figure 6);
- Modification of the dynamic properties of the pressure plate and rotor casing reduces the number and/or size of sudden changes in rotor speed in the resonance range.

The variable pressing load between the driving belt and the rotor pin is caused by the oscillation of the belt. In general, the reasons for belt oscillation are wear, insufficient belt tension, and changes in ambient temperature. The measurement was at a constant ambient temperature. A certain temperature change in the body can occur due to friction on the belt-rotor pin during a sudden change in speed. The measuring belt had no signs of wear, and the appropriate belt tension had been set by separate measurement. Thus, the dynamic and modal properties of the excited components, depending on mass, stiffness, damping, natural frequency, and natural modes, are decisive for the fluctuating pressing load and effects on the rotor within the resonance range of the rotational speed.

### 3.2. Components Suitable for Redesign

In general, a machine tool mechanical system is a damped mass-spring system driven by an external periodic excitation force. The forced vibrations of corresponding amplitudes and frequencies are the response. While increasing the operating speed, the displacement response of a forced mass-spring system changes, and resonance can appear. In order to be able to operate the system, despite the phenomenon of resonance, we can (i) change the operating speed of equipment, (ii) increase damping, or (iii) change natural frequency, and thus shift the resonance peak to the right or to the left using quantities of mass and stiffness according to the well-known formula

(3) $$\Omega_{n}=\sqrt{\frac{k}{m}}$$

where *Ω*_n_ is the undamped natural frequency, *k* is the stiffness, and *m* is the mass of the mechanical system (equipment).

Vibration amplitudes can be reduced in the low-frequency region by increasing the stiffness and in the high-frequency region by increasing the mass of the system. However, such an approach is traditional. A modern approach is the use of materials of high damping capacity and at the same time of light-weight and high stiffness, possibly even strength. It is an unusual combination of properties that is able to be obtained from modern advanced materials. In the region of resonance peak, the vibration amplitudes can be reduced only by increasing the damping of the system.

Therefore, we decided to solve the problem by preserving the original design with the standard materials of steel and aluminum alloy and supplementing the mechanical system with modern materials characterized by high material damping and increased stiffness. Thus, the resonance peak is lowered and slightly shifted to the right. Moreover, the original vibration amplitudes in the low-frequency region are also lowered and the driving frequency can be increased. The materials for that redesign are listed in Table 1 and Table 2. In addition, reducing the displacement response of the resonance peak is only possible by increasing the damping of the system. The damped natural frequency, *Ω*_d,_ of the mechanical system (equipment) is

(4) $$\Omega_{d}=\Omega_{n}\sqrt{1-\zeta^{2}}$$

where ζ is the damping ratio.

We selected components suitable for redesign according to their shape and location in the assembly. These were either the components containing cavities or the space between components in the assembly that creates a cavity. The components, namely pressure plate PP and rotor casing RC, were determined for redesign according to Figure 3 and Figure 4.

### 3.3. Dynamic Response of Machine Mechanical System with Redesigned Components

**Figure 8:**
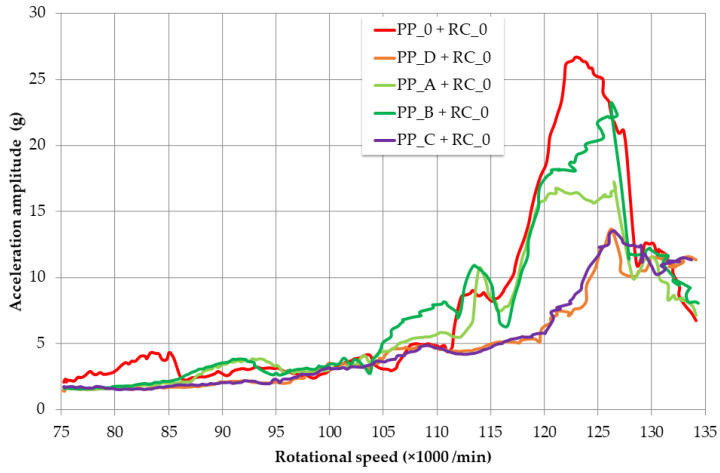
ACC amplitude vs. rotational speed curves obtained during increase in rotor revolutions, with torsional vibrations in resonance, from 75,000 to 135,000 min^−1^ over 80 s.

We performed measurements for different types of redesigned pressure plates based on polymers and carbon fibers. The influence of these pressure plates, compared to the original state, on the system vibrations resulting from rotor revolutions during their continuous increase from 75,000 min^−1^ to 135,000 min^−1^ over 120 s is shown in Figure 8. Pressure Plates A–D have almost the same mass (the 4.6% maximum difference mentioned earlier), which is very small compared to the mass of the system. The influence of the mass parameter on the change in dynamic response is negligible. The original maximum amplitude of the resonance peak (red in Figure 8) is slightly shifted to the right by increasing the stiffness of the system and is reduced due to the use of highly damping composite polymers. Increasing the stiffness and the damping of the system are parameters that positively affect the displacement response of the described system. The improvement in stiffness of Pressure Plates A–D is about 50%, compared to the original. Estimation of the stiffness increase was made by numerical analysis. Young’s modulus of elasticity of Pressure Plates A and B and for C and D for that analysis was determined by the rule of mixtures and from technical list data, respectively.

As the percentage of nanofibers in PP_A and PP_B was the same, 1 wt%, we assume that the different response lies in the different aspect ratios of the MWCNTs. PP_C and PP_D had a very similar response. The addition of continuous aramid and carbon fibers to the structure does not have a significant effect; the dominant effect is the polymer matrix with short carbon fibers and also the shape of the passive damper.

**Figure 9:**
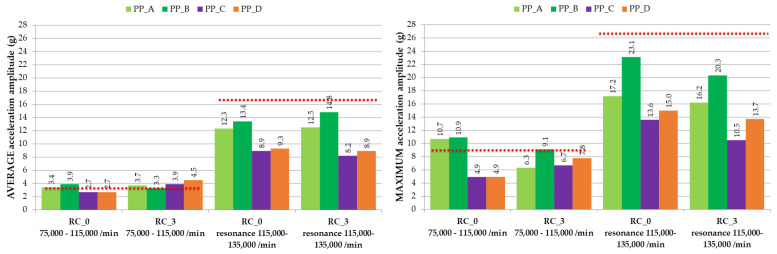
Average and maximum ACC amplitudes of redesigned pressure plates and rotor casings; red dotted line is the average and maximum acceleration amplitudes for PP_0 + RC_0.

For evaluation, the graph of ACC amplitude in response to increasing operating speed was divided into two ranges, namely Low-Frequency Region 1 (75,000–115,000 min^−1^) and Resonance Peak Region 2 (115,000–135,000 min^−1^). Average and maximum ACC amplitudes were measured for individual pressure plates and rotor casing RC_0. The same measurement was also carried out with the redesigned RC_3. The results are shown in Figure 9 and Table 4.

In Low-Frequency Region 1, there are no significant changes in vibration amplitude response caused by redesign, which is in accordance with the principles of machine dynamics. However, changes in the area of the resonance peak, i.e., in Region 2, are significant. Average values of ACC amplitude were reduced by rotor casing RC_0, and pressure plates, PP_A and B or PP_C and D, by an average of 25.0% and 44.5%, and reduced by RC_3 and PP_A and B or PP_C and D by 16.8% and 47.9%, respectively (Figure 9, Table 4). Pressure plates PP_C and PP_D are more preferable, and PP_C has a slightly better reduction of average amplitudes (8.9 g and 8.2 g) compared to PP_D (9.3 g and 8.9 g). We assume that this is due to the presence of aramid fiber, which is characterized by a higher damping parameter tan delta (according to [25]). As for the maximum values of the ACC amplitudes, compared to the original design, maximum values are reduced by 35.5% and 43.2% for RC_0 and RC_3, respectively. There, RC_3 has a slight advantage, because it reduced the maximum amplitudes slightly more than RC_0. When comparing average values for RC_0 with those of RC_3, RC_3 does have a slight advantage over RC_0. It follows that RC_3 does not have a significantly different resonance response, in the case of torsional oscillations, compared to RC_0. A significantly greater effect of RC_3 has been observed in reducing the amplitudes of translational vibrations [26].

### 3.4. Acoustic Emission

We evaluated the acoustic emission of the system at a constant operation speed of 135,000 min^−1^ (Table 5). With rotor casing RC_3, the acoustic emission has slightly lower values for each modified pressure plate. However, we can state that using the redesigned PP and RC components, the acoustic emission is without significant change at resonance with torsional oscillations.

## 4. Discussion

Polymers, as the matrix material for all tested PPs, are characterized by a long-chain molecular structure [27]. This fiber molecular structure is enriched by a filler of nanotubes (PP_A and PP_B) and chopped fibers (PP_C and PP_D). Moreover, pressure plates, PP_C and PP_D, contain continuous fibers of aramid and carbon, respectively, in layers along their outer edge. The described structures are of a hierarchical design at the macro-, micro, and nanoscale.

**Figure 10:**
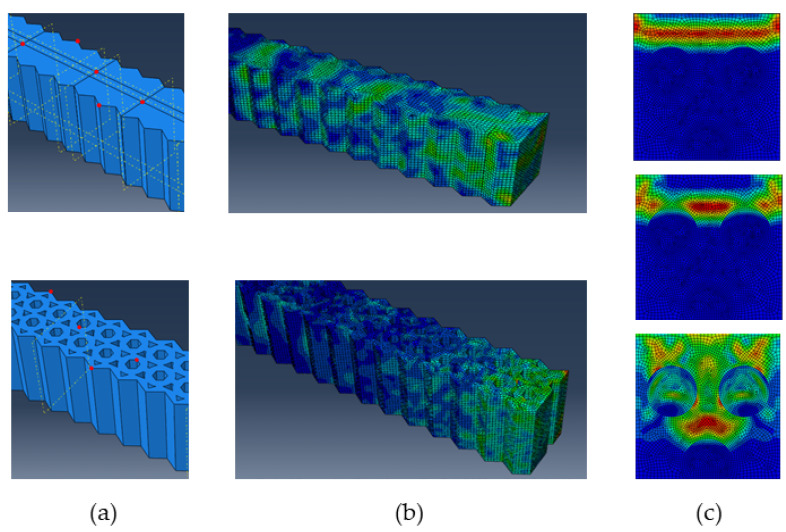
Stress wave propagation: (**a**) CAD models without (upper) and with holes (lower); (**b**) dispersed stress wave in those two models; (**c**) stages of stress wave propagation in composite material with reinforcing elements, initial phase (upper), and following phases (middle, lower).

Numerical analysis can show the influence of the component’s macroshape and the internal structure of the material as sources of improved performance of the component. This finite element analysis was created in the Abaqus/Explicit software intended for transient dynamic events that take place in very short time periods (more in [28,29]). The goal was to visualize the propagation and dispersion of the stress wave at the macro- and microscale and thus identify the sources of increased damping by the pressure plates. A simulated additional material of pressure plates PP_C and PP_D was excited by an impulse force at the free right end with all degrees of freedom removed at the other (left) end (Figure 10b). The explicit dynamics procedure is split into many small time increments to integrate through time. The impulse of force lasts 2 × 10^−6^ s with its maximum in time 10^−6^ s. The fine finite element mesh consisted of 116.653 solid finite elements, i.e., bricks C3D8R.

We compared the responses for two different macrogeometries of additional material, i.e., with and without holes (Figure 10a). The inner macroscopic structure, with holes, of PP_C and PP_D is shown in the lower image in Figure 10a. The dispersed stress wave evoked by the unit impulse force representing one oscillation is shown in Figure 10b. The upper image is of an additional material without any holes, and the lower image is of an internal structure with hexagonal and triangular holes corresponding to the real structure. The size and duration of the impulse and the pulse energy are equal for both. The additional material with the internal macroscopic structure was able to absorb the energy of the impulse with its front part only. In contrast, at the same time, in the solid material without holes, the stress wave had no obstacles in its propagation throughout the entire volume, and it could continue farther into the other machine structure (Figure 10b, upper). Figure 10c illustrates the propagation and dispersion of a stress wave at the microscale. An analyzed unit cell contains three reinforcing elements (fibers or particles). For a 2D model, these are represented by three circles, which are obstacles to the propagation of the wave, and thus they contribute significantly to the dispersion and absorption of the stress wave. Figure 10c, upper, shows how a stress wave propagates in a homogeneous matrix. Furthermore, Figure 10c, middle and lower, shows how the stress wave gradually hits the fibers and is thus rapidly dispersed and absorbed, and the favorable macroscopic response is obtained.

## 5. Conclusions

The aim of the study was to reduce or eliminate cage slippage and torsional vibrations in the resonance by changing the dynamic properties of the pressure plates and/or rotor casing of a high-speed rotor bearing. The reduction in kinematic slippage is necessary to ensure the safe and not shortened service life of the rotor bearing. We compared five types of pressure plates for damping an idler pulley operation and two rotor-bearing casings. We evaluated the dynamic response of the machine tool unit during the operation of the rotor bearing by measuring and evaluating vibrations up to 60 kHz. We used a high-frequency textile rotor bearing with torsional vibrations in resonance. The effect of torsional vibrations does not occur in resonance with all rotor bearings, but if it did occur during operation, the rotor bearing underwent unstable operation in resonance conditions with harmful and undesirable torsional impulse vibrations at significant amplitudes, resulting in a rapid shortening of the service life.

Modification of specific components with the help of composite material filling has a significant impact on the dynamic properties and dynamic response of the measured machine tool unit. Amplitudes of rotor-bearing torsional oscillations are reduced by reducing the amplitude of the idler pulley with pressure plates and also by modification of the rotor casing using stiffness and mainly passive damping of polymer composite materials. In the tested mechanical system, the influence of the pressure plate on reducing the amplitude of torsional vibrations at high rotational resonance speeds is more significant than that of the rotor casing. A significantly greater effect of rotor casing RC_3 in reducing the translational vibrations of lower amplitudes has been shown in a previous study [26].

From the analysis of the measured data on the performance of the redesigned pressure plates and rotor bearing, the following can be concluded:

- The PP_A and PP_B pressure plates containing nanofibers in composite in three macrolayers reduce the average and maximum vibrations of the original rotor casing, RC_0 by 25.0% and 24.5% and for RC_3 by 16.8% and 31.7%, respectively.
- Pressure plates PP_C and PP_D, characterized by a specific macroshape and a hierarchical carbon-based structure, significantly reduce amplitudes. They are suitable for combination with the original rotor casing, RC_0, which can achieve a reduction of average and maximum amplitudes by 44.5% and 46.4%, respectively. Their combination with rotor casing RC_3 filled by polymer–concrete is slightly better; the reduction in average and maximum amplitudes by this combination is 47.9% and 54.7%, respectively.

The influence of the macroshape and internal structure of these structures was shown using stress wave fields and numerical simulation. The modified pressure plates are based on either nanofibers or microfibers of carbon, have different macroshapes, and have an internal hierarchical structure. The additional material for component modification has high material damping properties and increases stiffness; moreover, the honeycomb shape with its internal cavities contributes to the fast damping of mechanical waves. The increase in stiffness and mass of each of the modified plates is in the range of 5%.

Further long-term tests on a real production line outside the test bench should be the next phase of testing.

## Figures and Tables

**Table 1:** Pressure plates and their description.

	Matrix	Fibers	wt%	Fiber Aspect Ratio from up to		Mass (Grams)
PP_0 (original)	-	-	-	-	-	19.7
PP_A	Epoxy resin L 285	MWCNT ø = 6–13 nm, *l* = 2.5–20 µm	1.0	1:192 13 nm:2.5 μm	1:3333 6 nm:20 μm	36.0 *
PP_B	Epoxy resin L 285	MWCNT ø = 110–170 nm, *l* = 5–9 µm	1.0	1:29 170 nm:5 μm	1:82 110 nm:9 μm	35.1 *
				**Fiber type**	**Layers**	
PP_C	Nylon	Carbon/Aramid	-	Short/Continuous	13	36.8 *
PP_D	Nylon	Carbon/Carbon	-	Short/Continuous	11	36.8 *

* mass without original steel pressure plate.

**Table 2:** Basic mechanical parameters.

	Tensile Modulus (GPa)	Tensile Strength (MPa)	Tensile Stress at Yield (MPa)	Flexural Strength (MPa)	Compressive Strength (MPa)	Density (g/cm^3^)
Epoxy resin	3.1	72	-	-	140	1.15
Onyx	2.4	-	40	71	-	1.2
Continuous Kevlar fiber	27	610	-	240	-	1.2
Continuous Carbon fiber	60	800	-	540	-	1.4

**Table 3:** Rotor casings and their description.

	Matrix	Filler	wt%	Grain Size (mm)	Additional Mass (Grams)	Mass (Grams)
RC_0 (original)	-	-	-	-	-	98.0
RC_3	Epoxy resin L 285	Silica sand	70.0	0.3–0.6	50.7	148.7

**Table 4:** Comparison of average and maximum ACC amplitudes in the resonance range.

Resonance Range 115,000–135,000 min^−1^ *								
	**RC_0**							
	**Average**				**Maximum**			
Pressure plate	A	B	C	D	A	B	C	D
ACC amplitude (g)	12.3	13.4	8.9	9.3	17.2	23.1	13.6	15.0
Average (g)	12.85		9.1		20.15		14.30	
Decrease compared to PP_0 + RC_0 (%)	25.0%		44.5%		24.5%		46.4%	
Average (g)	10.975				17.23			
Decrease compared to PP_0 + RC_0 (%)	33.1%				35.5%			
	**RC_3**							
	**Average**				**Maximum**			
Pressure plate	A	B	C	D	A	B	C	D
ACC amplitude (g)	12.5	14.8	8.2	8.9	16.2	20.3	10.5	13.7
Average (g)	13.65		8.55		18.25		12.10	
Decrease compared to PP_0 + RC_0 (%)	16.8%		47.9%		31.7%		54.7%	
Average (g)	11.100				15.175			
Decrease compared to PP_0 + RC_0 (%)	32.3%				43.2%			

* Within resonance range, the average and maximum acceleration amplitudes for PP_0 + RC_0 were 16.4 g and 26.7 g, respectively.

**Table 5:** Acoustic emission (AE) at 135,000 min^−1^ for a rotor bearing with torsional oscillations in resonance.

PP	0	A	B	C	D
AE (dB rms)					
RC_0	97.4	102.6	101.9	105.1	105.3
RC_3	102.8	99.4	98.7	101.0	101.2

## Data Availability

Not applicable.
